# Comparative evaluation of fracture resistance force of three different posterior aesthetic crowns and occlusal wear of antagonist primary teeth – An *in vitro* study

**DOI:** 10.4317/jced.62730

**Published:** 2025-05-01

**Authors:** Pooja V. Ravi, Kavitha Ramar, Víctor Samuel, Pradeep K. Yadalam, Carlos M. Ardila

**Affiliations:** 1 Department of Pediatric and Preventive dentistry, Saveetha Dental College and Hospitals, Saveetha Institute of Medical and Technical Sciences, Saveetha University, Chennai, Tamil Nadu, India; 2 Department of Pediatric and Preventive Dentistry; SRM Kattankulathur dental college and hospitals, SRM institute of science and technology. SRM Nagar, Kattankulathur, Kanchipuram, Tamil Nadu, India; 3 Department of Periodontics, Saveetha Dental College, Saveetha Institute of Medical and Technology Sciences, SIMATS, Saveetha. University, Chennai, Tamil Nadu, India; 4 Department of Basic Sciences, Biomedical Stomatology Research group, Faculty of Dentistry, Universidad de Antioquia, U de A, Medellín, Colombia

## Abstract

**Background:**

Aesthetic restorations for managing severely carious primary molars pose a clinical challenge, particularly regarding limited treatment options and the importance of aesthetics to parents. While zirconia crowns are commonly used for posterior teeth due to their aesthetic appeal, advancements in digital technology have introduced alternative options such as polymethylmethacrylate (PMMA) crowns and 3D printable photopolymer resin crowns, which offer cost-effective alternatives. This in vitro study aimed to comparatively evaluate the fracture resistance force (FRF) and occlusal wear of three types of posterior aesthetic crowns: milled zirconia crowns, PMMA crowns, and 3D printable photopolymer resin crowns.

**Material and Methods:**

A universal mechanical testing machine was employed to measure the FRF, while occlusal wear was assessed by subjecting five samples from each group to chewing simulation with opposing natural teeth. Preoperative and post-operative 3D scan measurements were obtained.

**Results:**

The average force required to fracture 3D printable photopolymer resin crowns was 877.212 N, compared to 1,326.522 N for PMMA crowns and 1,712.488 N for milled zirconia crowns. Significant differences in occlusal wear were observed in the zirconia (*p*-value= 0.005) and 3D printed crown groups (*p*-value= 0.010) when comparing pre-operative and post-operative measurements.

**Conclusions:**

This study suggests that PMMA crowns exhibit clinically acceptable fracture resistance force and cause minimal occlusal wear on opposing primary teeth. These findings support the potential long-term clinical use of PMMA crowns as an alternative to zirconia crowns in aesthetic restorations for severely decayed primary molars.

** Key words:**Zirconia crowns, polymethylmethacrylate crowns, Photopolymer resin crowns, Primary teeth.

## Introduction

Pediatric dentistry has been greatly impacted by the need for aesthetic appeal. Under clinical circumstances involving extensive caries, developmental defects, or multiple decayed surfaces, the American Association of Pediatric Dentistry (AAPD) and the British Society for Pediatric Dentistry recommend the use of restorations that fully cover the visible part of the tooth (1). Stainless steel crowns (SSCs) are considered to be the standard for restoring primary teeth with extensive damage and endodontically treated primary molars (2). SSCs do not fully satisfy the aesthetic preferences of modern-day parents, who frequently exert considerable influence over the decisions regarding dental restorations for their children (3,4). Consequently, there has been a surge in the development of alternative restorative options for children that aim to fulfil aesthetic desires, such as pre-veneered SSCs, composite facing SSCs, polycarboxylate crowns, shell crowns, composite crowns, celluloid crowns, and zirconia crowns (ZR) (5,6). Due to the absence of a metallic basis, ZR crowns are a newer alternative to previously fabricated SSCs, with superior aesthetics. Zirconia, a crystalline zirconium dioxide, outperforms traditional dental restorations in terms of mechanical and fracture strength as well as chemical and volume stability (7). Consequently, it is the perfect material for creating anatomically shaped crowns and offers the best possible tooth-like appearance (8).

ZR crowns have remarkable shortcomings, including increased thickness that necessitates more tooth reduction, the potential for pulp exposure, the requirement for passive seating during cementation, and the inability to adjust the crown to fit the tooth (9). One of the significant disadvantages of ZR crowns is their strong abrasive properties causing opposing natural enamel to be worn away during occlusal interactions. The limited evidence on their long-term effectiveness and high cost have significantly hindered their widespread use, particularly among economically disadvantaged children who experience higher rates of tooth decay (10).

Therefore, continuous efforts to create primary tooth restorations that are both aesthetically pleasing and capable of solving the issues with earlier crowns, lead to the notion of using the long-term temporization concept in primary teeth. Provisional crowns can currently be built out of a range of materials, starting with conventional acrylics made of methyl methacrylate polymers, which have the advantages of reduced wear from opposing tooth enamel, the ability to absorb functional stresses due to their low modulus of elasticity, and is less invasive (11,12). Temporary or permanent prostheses for primary teeth can now be produced using 3D printing technology and printable materials, offering durability against occlusal stress and chemical reactions in the oral environment while being cost-effective (13). Additionally, one notable downside of ZR crowns is their abrasive nature, which leads to considerable wear on opposing natural enamel during occlusal interactions. Different materials have varying histories regarding crown durability (14). Unfortunately, obtaining clinical evidence of enamel wear is challenging, but *in vitro* research can provide valuable insights.

Hence, this study aims to conduct an *in vitro* evaluation of three different posterior aesthetic crowns, focusing on their fracture resistance force and occlusal wear on opposing primary teeth. The tested crowns include milled zirconia crowns, polymethylmethacrylate (PMMA) crowns, and 3D printable photopolymer resin crowns. The findings of this research will contribute to understanding the long-term clinical use and potential advantages of these aesthetic crown options for restoring severely decayed primary molars.

## Material and Methods

The current study was a prospective observational study. The study design was approved by the Institutional Scientific and Ethical Review Committee (IEC approval 8403/IEC/2022) before the commencement of the study. G Power version 3.1.9.2 software was used to compute the sample size; the result was a power of 0.95. Six samples were needed for each group. The current study proceeded with a sample size of 10 per group making a total sample of 30. The thirty samples were divided into the following three groups: Group A - milled zirconia crowns (n=10), Group B- PMMA crown (n=10), and Group C- 3D printable photopolymer resin crowns (n=10]. Thirty positive replicas of the primary posterior teeth were obtained using Interacrylic Ortho Resin from rubber moulds and were allowed to be set for 24 hours. Subsequently, thirty crown replicas were randomly assigned to three groups, each containing 10 crowns. Tooth preparation was done depending on the groups.

Tooth preparation for zirconia crown: the occlusal surface reduction of 1.5 to 2 mm with a rough, long-tapered diamond bur was done, followed by a 15 to 20 percent circumferential reduction, and a complete subgingival reduction with a feather-edged border, roughly 1.5 mm deep was done.

Tooth preparation for PMMA and photopolymer resin crowns: using a diamond round end taper bur, buccal, lingual, mesial, and distal walls were prepared for 0.8 to 1.0 mm. Then, with a wheel no. 909 bur a convergence angle of six degrees was made, followed by circumferential chamfer the margin preparation and reduction of the occlusal surface by 1.0 to 1.5 mm was done (15).

**Figure 1:**
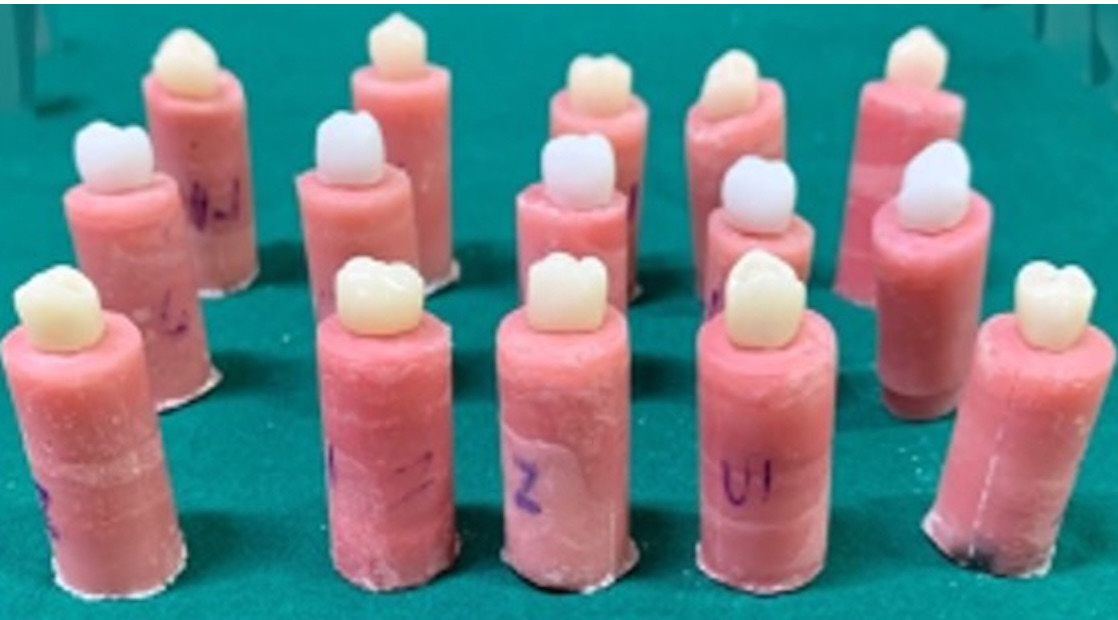
Cementation of fabricated crowns.

Digital scanning of all the prepared samples was done using EXOCAD version 3.0 software and standardization was done for each sample in their respective groups. A composite finishing bur was used to remove any undercuts in the prepared teeth that were evident. Exocad GmbH software (GmbH, Darmstadt, Germany) was used to design the crowns for full coronal restoration as a restoration type, creating Standard Tessellation Language (STL) files and fabricating the crowns in accordance with assigned groups. Group A crowns were made using Ceramill Zolid ht plus zirconia discs, Group B- PMMA crowns were made using Ceramill temp PMMA discs by Computer-Aided-Designing \ Computer-Assisted-Manufactuering- technique, Group C crowns were made using formlabs three Dimensional prinTable, biocompatible, photopolymer resin crowns fabricated via 3D dental printer DLP printing type ( Table 1). The fabricated crowns were trial-checked to ensure a passive fit. All the fabricated crowns were luted using glass ionomer cement, according to the manufacturer’s guidance, and then the die–crown units were allowed to be set for the next 24 hours (Fig. 1). After crown cementation samples in each group were subdivided according to the test performed.

-Evaluation of Fracture Resistance Force (FRF) of three different esthetic crowns 

**Figure 2:**
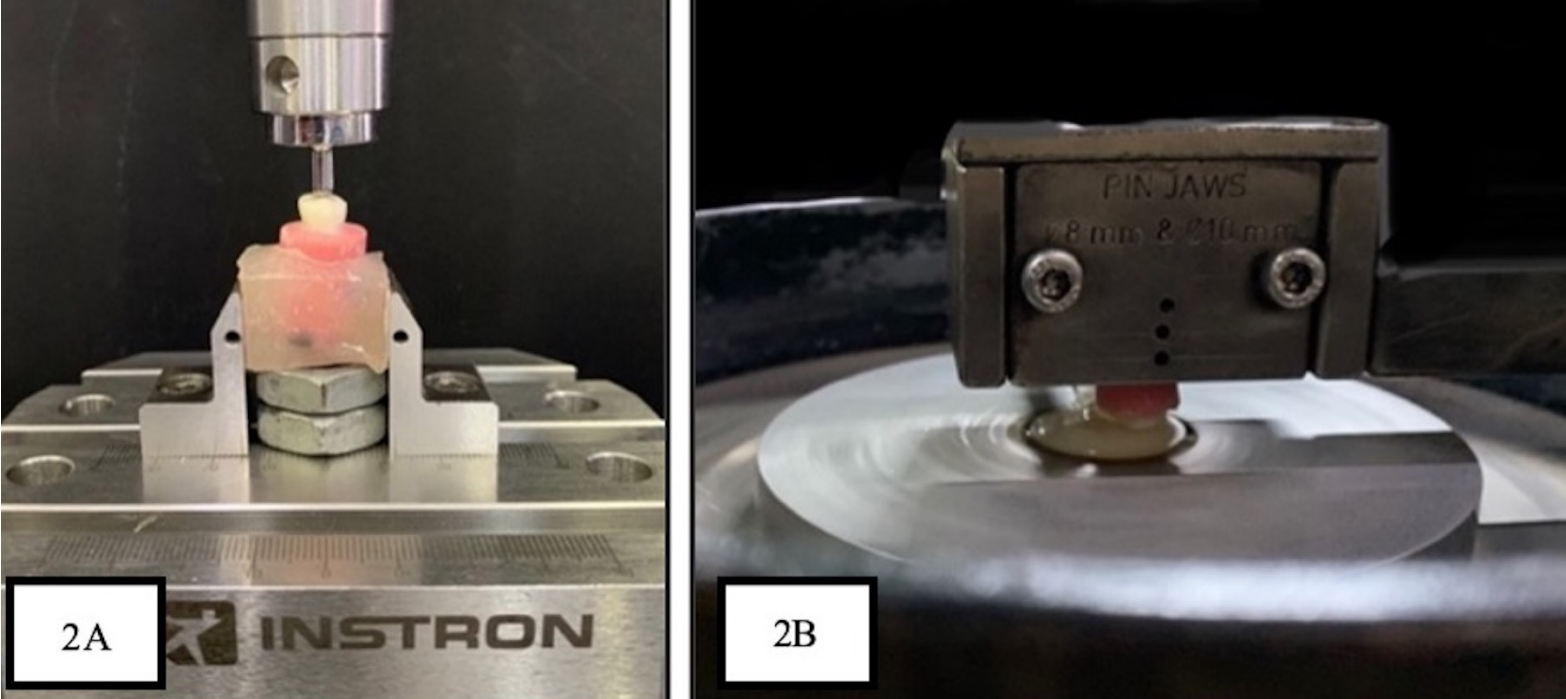
A- Samples mounted on universal testing machine. B- Samples subjected to wear analysis in pin on disc tester.

**Figure 3:**
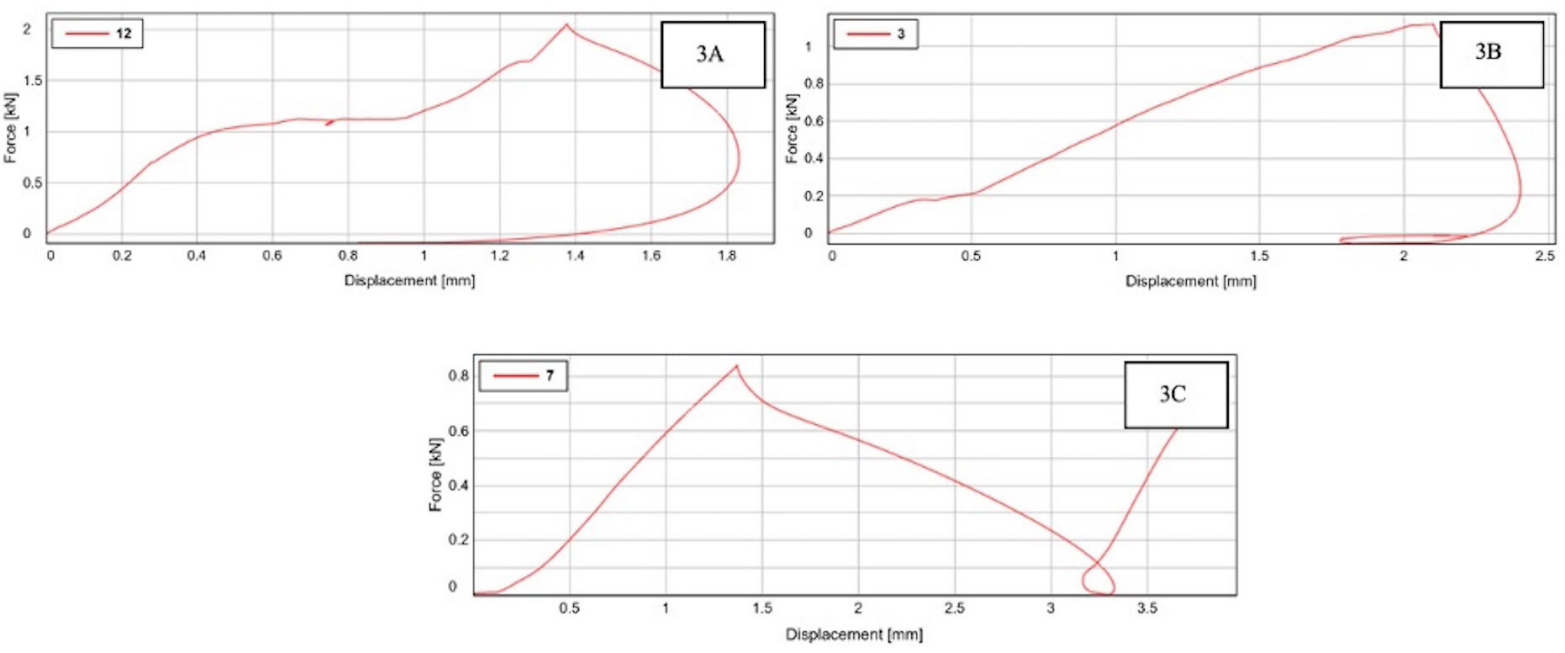
Graphical representation of fracture resistance force values. A- zirconia crown group. B- Polymethylmethacrylate crown group. C and D printed crown group.

Fifteen samples (five samples from each group) that received full coronal restoration were randomly selected to evaluate the FRF. Evaluation of Fracture Resistance Force was performed with an Instron universal mechanical testing machine (Fig. 2A). To simulate a cusp contact, the uniaxial force was applied through a stainless-steel ball fixture. The experiment was run in a single cycle, keeping the crosshead’s speed at 1 m/min, until the experiment’s crowned teeth fractured. The values were then recorded for statistical analysis. The graphical representation of FRF values is represented in Figure 3.

Evaluation of occlusal wear of opposing primary teeth

Based on the predetermined inclusion and exclusion criteria, fifteen freshly extracted human primary first and second molars were collected ( Table 2).

**Figure 4:**
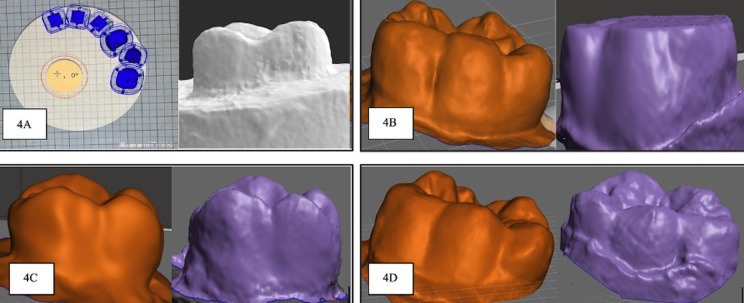
A- Digital scanning of prepared tooth. B- Pre-operative and post-operative scanned images of antagonist teeth subjected to wear test against zirconia crown. C- Pre-operative and post-operative scanned images of antagonist teeth subjected to wear test against polymethylmethacrylate crown. D- Pre-operative and post-operative scanned images of antagonist teeth subjected to wear test against 3D printed crown.

The teeth were rinsed to remove soft deposits and tissue fragments. Using visible light, teeth were inspected for soundness and fractures. The collected teeth samples were stored in the artificial saliva in a sterile container. Preoperatively each tooth sample was subjected to 3D scanning using EXOCAD version 3.0 software. Consequently, five samples for each group were subjected to artificial chewing stimulation with opposing prepared die-crown models for 1,20,000 chewing cycles using a dual-axis and a 50 Newton masticating force (Fig. 2B). They were continuously replenished with artificial saliva. After the chewing simulation, the samples were washed, dried, and subjected to a post-operative 3D scan using EXOCAD version 3.0 software (Fig. 4A). The pre-operative and post-operative scanned STL files were converted to JEPEG format and imported to Autodesk, Meshmixer software (version 3.5). The imported pre-operative and post-operative images were analyzed to calculate the wear (Fig. 4B,C,D). The calculated values were subjected to statistical analysis. All three types of tested crowns were assessed and compared for the force required for fracture and the amount of occlusal wear of natural teeth was assessed and compared using statistical analysis.

-Statistical analysis

The data collected was quantitative and was subjected to Normality testing using Shapiro Wilk’s test and the data seemed to be normally distributed, hence parametric tests of significance were used. The mean values were depicted as mean and standard deviation and intergroup comparisons were made by ANOVA (Analysis of variance) and intra-group analysis was made using repeated measures ANOVA. A *p-value* of <0.05 was considered to be statistically significant.

## Results

The mean values of fracture resistance were 1712.48 ± 268.47 in group A, 1326.52 ± 307.75 in group B, and 877.21 ± 61.77 in group C. The mean values of fracture resistance between the groups were statistically significant (F = 15.36, *p* <0.001).

A highly statistically significant difference in tooth wear was noted in the zirconia group (*p-value*= 0.005) and a significant difference was seen in the 3D printed crown group (*p-value*= 0.010). The mean values of the study parameters among the groups are presented in  Table 3. Inter-group comparisons are presented in  Table 4.

## Discussion

The advancements in bonded cosmetic restorations have sparked interest in exploring full-coverage restorative materials that can provide both aesthetics and durability in the pediatric population (16). With recent breakthroughs in digital dentistry, 3D-printed photopolymer resin offers the advantage of customization, the creation of crowns that perfectly match the patient’s morphology and aesthetically pleasing crowns. Similarly, with the application of CAD/CAM technology, crowns with perfect occlusal anatomy and proximal contact sites with optimal marginal adaptability can be produced. To date, zirconia crowns are the only aesthetic choice of full coronal restoration for posterior teeth in primary dentition. It is well recognized that different materials have varying degrees of durability for crowns depending upon the occlusion and continual biting forces. The choice of crown material must be made with care, especially when working with primary teeth because they are more susceptible to fracture and wear. Hence, this study sought to investigate the effectiveness of 3D-printed resin crowns compared to polymethyl methacrylate [PMMA] crowns and milled zirconia crowns in terms of FRF and wear of antagonist natural teeth. The finding of our study reveals, the average force required to fracture a crown made of 3D prinTable photopolymer resin was 877.212 N. PMMA crowns made from milled blocks using computer-aided design/computer-aided manufacturing (CAD/CAM) technology demonstrated higher fracture resistance (1,326.522 N) compared to 3D-printed resin crowns, placing them on par with zirconia crowns (1,712.488 N). This is attributed to the high-pressure and high-temperature polymerization process involved in creating milled PMMA blocks reduces manufacturing flaws and improves fracture toughness (12).

Similar findings in 1993 by Braun *et al*. inferred that the “maximum bite force increased from 78 N at 6-8 years to 176 N at 18-20 years” in the region of the primary molars (17,18). According to Owais *et al*., “the maximum biting force increased from 176 N in the early primary stage to 433 N in the late mixed stage”(19).

With our study result, we infer that the FRF values of all experimental crowns exceeded the average biting forces produced by different age groups, indicating their potential durability in clinical situations.

A restorative dental material, according to Seghi, should have a “wear degree comparable to that of the enamel” (20). Wear occurs as a complex form of chemical and mechanical wear. The wear of the crown material itself and the wear of opposing natural teeth are important issues that should be taken into account when choosing crowns in clinical practice (21,22).

The results of this study demonstrate that zirconia crowns exhibited noticeable wear (*p-value*= 0.005) on antagonist teeth compared to PMMA and 3D-printed crowns. The overall volumetric antagonistic enamel loss in this study’s ZR group (2.26) is comparable to that reported by Jung *et al*. (23,24).

Chewing simulators are commonly used to simulate oral conditions, generating forces within the range of 20 to 120 N, which is clinically replicable (25). In this study, a chewing force of 50 N, representing the mean value of physiological biting forces, was applied (26). The number of chewing cycles in a simulator is often associated with wear, and a range of 100,000 to 120,000 cycles corresponds to approximately six months in an individual (27). Non-standardized enamel cusps were used as antagonists in this study, as standardized enamel removes the aprismatic enamel layer, which is believed to be more resistant to wear than prismatic enamel (28). Future studies could consider increasing the time duration to measure tooth wear beyond six months.

The innovation in this study lies in the incorporation of two distinct elements: the utilization of 3D prinTable photopolymer resin and the comparison with milled crowns. Examining its application in restoring primary molars within pediatric dentistry can contribute an additional dimension to the management of primary dentition. This is particularly noteworthy as 3D printing demonstrates significant potential in the realm of clinical dentistry, spanning across various disciplines.

Another noteworthy aspect of this study is the adoption of a 3D-scanning methodology, offering a more precise approach compared to conventional methods. To our knowledge, there is currently no published literature assessing the wear of opposing teeth in the presence of 3D-printed photopolymer resin crowns using a chewing simulator. An additional distinctive feature of this research is the consideration of a natural lubricant, saliva, to accurately ascertain the variations in wear between opposing natural teeth and the crown material.

## Conclusions

The 3D-printed resin crowns exhibited fracture resistance comparable to both PMMA and milled zirconia crowns. Furthermore, the wear on antagonist teeth was noticeably reduced with PMMA and 3D-printed crowns compared to their zirconia counterparts. Our hypothesis posits that 3D-printed resin crowns, along with PMMA crowns, have the potential to yield long-term restorations suiTable for intraoral use, boasting sufficient mechanical properties. These crowns not only demonstrate durability but also present an aesthetically pleasing alternative to zirconia crowns, addressing concerns related to wear on antagonist teeth and manufacturing costs. In conclusion, the findings support the viability of 3D-printed resin and PMMA crowns as effective and durable solutions in restorative dentistry.

## Figures and Tables

**Table 1:** Materials used in the study.

Crowns	Materials	Manufacturers
Zirconia crowns	Ceramill Zolid ht plus zirconia discs	AMANN GIRRBACH AG, Herrschaftswieses 1, Koblach, Austria
Polymethylmethacrylate crowns	Ceramill temp PMMA discs	AMANN GIRRBACH AG, Herrschaftswieses 1, Koblach, Austria
3D-printed photopolymer resin crowns	Formlabs photopolymer resin	Formlabs, Inc. Somerville, MA, USA

**Table 2:** Criteria for collection of natural teeth.

INCLUSION CRITERIA	EXCLUSION CRITERIA
Primary molar teeth were extracted due to preshedding mobility.	Teeth that are severely damaged due to caries, erosion, abrasion, or hypoplasia.
Primary molar teeth with or without resorption of roots.	Teeth with severely attrited coronal portion, abnormal morphology.
Primary molar teeth with no caries up to the cementoenamel junction.	Permanent teeth.

**Table 3:** Intra-group comparison of the mean values of the study parameters among the groups.

Groups	Parameters	Mean	Standard Deviation	p-value
ZR	Tooth Wear Pre-operative	4.8201	0.51937	0.005***
	Tooth Wear Post- operative	2.7636	0.98490	
PMMA	Tooth Wear Pre-operative	5.2004	0.41459	0.100
	Tooth Wear Post-operative	4.7407	0.42474	
3D	Tooth Wear Pre-operative	5.9225	0.41436	0.010*
	Tooth Wear Post-operative	4.9136	0.72603	

Test used: Repeated measures ANOVA *p<0.05 is statistically significant **p<0.01 is statistically highly significant ***p<0.001 is statistically very highly significant ZR= zirconia crowns PMMA= polymethylmethacrylate

**Table 4:** Intergroup Comparison of the mean values of the study parameters among the groups.

Inter-group comparison of the study parameters	F-value	p-value
Fracture Resistance	15.364	0.001***
Tooth Wear Pre-operative	7.668	0.007*
Tooth Wear Post-Operative	12.759	0.001***

Test used: One way - ANOVA *p<0.05 is statistically significant **p<0.01 is statistically highly significant ***p<0.001 is statistically very highly significant

## Data Availability

The datasets used and/or analyzed during the current study are available from the corresponding author.
